# Protein-glutaminase improves water-/oil-holding capacity and beany off-flavor profiles of plant-based meat analogs

**DOI:** 10.1371/journal.pone.0294637

**Published:** 2023-12-06

**Authors:** Kiyota Sakai, Masamichí Okada, Shotaro Yamaguchi

**Affiliations:** Amano Enzyme Inc. Innovation Center, Kakamigahara, Japan; Universidad Tecnica de Ambato, ECUADOR

## Abstract

An unresolved challenge for plant-based meat analogs (PBMAs) is their lack of juiciness. Saturated fats significantly contribute to the juiciness of PBMAs, but there are concerns about the undesirable health effects related to saturated fats; thus, demand for their replacement with vegetable unsaturated oils has increased. Although many food additives are used to reduce the leakage of unsaturated oils, this solution cannot meet the clean-label requirements that have been trending in recent years. In this study, we aimed to develop better consumer-acceptable methods using protein-glutaminase (PG) to improve the juiciness of PBMA patties to meet clean-label trends. We found no significant difference between the visual surface of control and PG-treated textured vegetable proteins (TVPs). However, the microstructure of PG-treated TVP had a more rounded shape than that of the control TVP as observed under a scanning electron microscope. After grilling process, the PBMA patties composed of PG-treated TVP showed significantly higher liquid-holding capacities (a juiciness indicator) than the control patties. This suggested that PG treatment could potentially produce PBMA patties with increased juiciness. Interestingly, after the PG-treated TVP underwent the wash process, we found that PG treatment of TVP easily reduced the various beany off-flavor compounds by 58–85%. Moreover, the results of the *in vitro* protein digestion test showed that the amounts of free amino nitrogen released from PBMA patties composed of PG-treated TVP were 1.5- and 1.7-fold higher than those from control patties in the gastric and intestinal phases, respectively. These findings indicate that PG treatment of TVP could enhance the physical, sensory, and nutritional properties of PBMA patties and meet the clean-label requirements.

## Introduction

The global population was 7.5 billion in 2017 and could grow to 8.5 and 10 billion by 2030 and 2050, respectively [1]. With this exponential growth, it is difficult to maintain protein production at a level capable of coping with this rapid increase in demand [2, 3]. In addition, excess consumption of animal meat increases the risk of heart disease, type 2 diabetes, cardiovascular complications, stroke, and some cancer types [3]. In contrast, numerous studies have reported that replacing animal protein sources with plant-based proteins has health benefits [3]. Therefore, consumers are demanding food manufacturers to develop better acceptable and preferred plant-based foods to address the imbalance between protein demand and supply, and positively impact human health [4].

Despite technological advancements, the appearance, flavor (smell and taste), mouthfeel, and nutrition of plant-based meat analogs (PBMA) cannot mimic animal-based products [5]. An unresolved challenge for PBMAs is their poor sensory properties, particularly their lack of juiciness. Animal fat contributes greatly to the juiciness of meat and meat products [6]; similarly, solid fats (e.g., coconut oil and cocoa oil) contribute to the fat-like texture and mouthfeel of PBMA products [5, 7]. Coconut oil is the vegetable fat commonly used to improve the juiciness of PBMA products. However, animal and plant-derived solid fats have high saturated fatty acid content, which can cause health-related issues [8]. The World Health Organization (WHO) recommends that saturated fats should not constitute more than 10% of the total fat content in the diet [9]. Coconut oil comprises approximately 92% saturated fats and 8% unsaturated fats, thus, does not adhere to the standards set by WHO [10]. Several food manufacturers, including those in the PBMA product industry, have made concerted efforts to replace saturated fats with unsaturated oils.

PBMA patties mainly comprise textured vegetable proteins (TVPs) that simulate the muscle fibrillar structure of animal meats [11]. TVPs are the main ingredient in PBMA patties and account for 25% of the total content [5, 7]. The lack of juiciness of PBMA patties is due to their inability to retain unsaturated oils in a liquid state at room temperature. To overcome this limitation, polysaccharides and proteins are used as binders (mostly in combination) to retain unsaturated vegetable oils such as canola, olive, and sunflower oils in the PBMA patties [5, 7, 12]. Soy protein, pea protein, wheat gluten, rice protein, methylcellulose, carrageenan, and modified starch are used as binders in PBMA patties [5]; they contribute to emulsion stability and reduce oil leakage and purge loss, although their potency is insufficient [13, 14]. The beany off-flavor compounds volatilized from the plant-derived proteins of the binders are another challenge that contributes to poor product acceptance. Additionally, consumers have recently become more aware of clean-label products and are demanding a reduction in food additives. Therefore, the scientific community and food industry are developing improved methods to increase the juiciness of PBMA patties using vegetable unsaturated oils.

Various enzymes have been used in food processing and ingredient production to enhance the physical, nutritional, and/or sensory properties of food [15]. Recently, food processing using enzyme catalysis has drawn attention as an attractive strategy to create clean-label foods because enzymes are not considered additives in the case they are denatured or inactivated in the finished food product [16–20]. Modification of TVP (the main ingredient of patties) by enzymes could be an attractive tool for increasing the functionality of PBMA products while meeting clean-label requirements. However, the effects of TVP modification by enzymes have not been extensively studied [5, 7]. Proteases, protein-modifying enzymes, are widely used in the food industry [21, 22], but their activity is difficult to control, which can increase the risk of developing peptide-derived bitter tastes or TVP collapse [23]. Protein-glutaminase (PG; EC 3.5.1.44) might be the only enzyme capable of modifying TVPs without these risks [24]. PG catalyzes the deamidation of glutamine residues into glutamate residues, modifying insoluble proteins via the negative charge repulsion of glutamate. Namely, protein modification by PG is able to be caused without proteolysis. To date, PG has been used to improve the functionality of soy, pea, chickpea, gluten, zein, oat, and coconut proteins [24–26]. Therefore, this study aimed to develop modification methods using PG to enhance the juiciness of PBMA patties while meeting clean-label requirements.

Our objectives were to investigate the effects of PG-catalyzed deamidation on the physical properties of soy protein isolate (SPI) and TVP. In particular, the liquid-holding capacity was examined as an indicator of the juiciness of PBMA patties and TVP. In addition, the effect of PG treatment on the beany off-flavor and the digestibility of PBMA patties were evaluated.

## Materials and methods

### Materials

TVP composed of soy protein were obtained from Marukome Co., Ltd. (Nagano, Japan). PG (Amano Enzyme Inc., Nagoya, Japan) is a commercially available food-grade product as protein-glutaminase 500. Hexanal, heptanal, octanal, nonanal, 2-octenal, 2-nonenal, benzaldehyde, 1-hexanol, and 1-octen-3-ol were purchased from FUJIFILM Wako Pure Chemical Corporation (Osaka, Japan).

### Enzyme assay

Soy protein isolate (SPI) and soy-based TVP were treated with 0–20 U/g-protein PG at 50°C for 180 min. The reaction was stopped by boiling the solution at 100°C for 5 min. The degree of deamidation (DD) was calculated as the ratio of the amount of ammonia released by the PG reaction to the total ammonia of glutamine or asparagine residues in protein molecules. The total ammonia amount was evaluated by measuring the amount of ammonia released from proteins deamidated by 3 N sulfuric acid. The ammonia amount released from protein molecules treated by PG or sulfuric acid was measured using a commercial kit (FUJIFILM, Wako Pure Chemical Corporation) following a previously described method [26].

### Degree of hydrolysis

The degree of hydrolysis was calculated as the ratio of soluble protein in the supernatant after the protein molecules were precipitated with 0.2 N trichloroacetic acid. The protein and peptide contents in supernatants were determined using the Kjeldahl method (N × 6.25).

### Water- and oil-holding capacities

The liquid-holding capacities (water- and oil-holding capacities) were evaluated [27]. Freeze-dried proteins were dissolved in deionized water or canola oil, and vortexed for 30 s. After incubation for 30 min at 25°C, the protein mixture was centrifuged at 2,000 × g at 25°C for 10 min. The precipitate and supernatant were weighed, and the liquid-holding capacities were calculated in grams of water or oil retained per gram of protein, respectively.

### Emulsifying activity and stability index

The emulsifying properties (emulsifying activity and stability) were evaluated [27]. Canola oil was mixed into a 1% protein solution at a final concentration of 25%. The protein and lipid mixture was homogenized at 10,000 rpm for 2 min and the resulting emulsion was collected after 0 and 10 min. Then, the homogenized emulsion was added to a 0.1% sodium dodecyl sulfate solution. The turbidity of the diluted solution was measured at 500 nm. The absorbance values measured at 0 min (A_0_) and at 10 min (A_10_) after homogenization were used to calculate the emulsifying activity and emulsion stability indices as follows:

emulsifying activity index (m^2^/g)  =  (2 × 2.303 × A_0_)/(F × protein weight)

emulsion stability index (%)  =  (A_10_ × *Δ*t)/(A_0_ –A_10_), where F is the oil volume fraction of 0.25, A_0_ and A_10_ are the absorbances at 0 10 min after homogenization, and *Δ*t  =  10 min.

### Foaming capacity and foam stability

The foaming properties (foaming activity and stability) were evaluated at 25°C [27]. A solution of the freeze-dried proteins (0.5% (w/v)) was prepared in deionized water. The foam was formed by homogenizing the protein solution at 18,000 rpm for 3 min. The homogenized samples were then transferred to a cylinder. The volume of the foam portion was recorded at 0 and 30 min to determine the foam activity and stability. These factors were evaluated using the following formulas:

foaming capacity (%)  =  [(VF_0_ − V)/V] × 100

foam stability (%)  =  [VF_30_/VF_0_] × 100 where V is the volume of the protein mixture before foam formation, VF_0_ and VF_30_ are the volumes of foam immediately after foam formation and after 30 min at 25°C, respectively.

### Preparation of PBMA patties

PBMA patties were prepared using the TVPs and a binder (methylcellulose), followed by the addition of water, canola oil, pea protein isolates, and potato starch. First, dried TVP (10 g) was immersed in water (15 mL) for 30 min for hydration, and 0–20 U/g-TVP PG was incubated at 50°C for 180 min. The hydrated TVP was mixed with 2.0% methylcellulose (final concentration). Subsequently, 5–25 g of water, 5–18 g of canola oil, and 1.0% potato starch were added to a 25 g wet-TVP. Then, the samples were blended for 60 s using a hand blender. The patty dough was molded (60 × 40 × 25 mm) and incubated at 60°C for 2 h. The dough was grilled at 150°C for 10 min and cooled to 20–25°C before further analysis. After the grilling process, PG activity in PBMA patties was completely undetected (data not shown).

### Scanning electron microscope (SEM) observation

Dried TVP (1.0 g) was immersed in water (1.5 mL) for 30 min for hydration, and 20 U/g-TVP PG was incubated at 50°C for 180 min. Control TVP was prepared under the same conditions, except PG was absent. High-resolution images of these TVP samples were obtained using an SEM (VHX-D500 microscope (Keyence Corp., Osaka, Japan)) working at 1.2 kV, according to a previous study [28]. In this study, sample observations were performed without metal sputtering treatment. Images were acquired at magnifications of 250× and higher.

### Measurements for texture profile analysis (TPA) of patties

TPA value was analyzed using a COMPAC-100II (Sun Scientific Co., Ltd., Tokyo, Japan) equipped with a cylindrical probe (31.4 mm^2^) as described in a previous study [29]. Hardness was evaluated as the maximum force recorded during the first compression. Cohesiveness and springiness were measured as the area of work and the distance moved, respectively, during the second compression divided by those of the first compression. Chewiness was calculated as hardness × cohesiveness × springiness.

### Cooking loss

The cooking-loss value was measured as the percentage weight difference between PBMA patties before and after grilling at 150°C for 10 min [30].

Cooking loss (%) = [(W_1_ –W_2_)/W_1_] × 100 where W_1_ and W_2_ are the weights of the patty before and after grilling (g).

### Headspace solid-phase microextraction-gas chromatography/mass spectrometry (HS-SPME-GC/MS)

The beany off-flavor compounds were analyzed using HS-SPME-GC/MS (Shimadzu, Kyoto, Japan) as described in a previous study [31]. 1,2-Dichlorobenzene (1.0 mg/g-patty) was used as an internal standard. Polydimethylsiloxane (Shimadzu, Kyoto, Japan) was used as the solid phase microextraction fiber for extraction, and volatile compounds were analyzed using a gas chromatography system (GC-2030, Shimadzu, Kyoto, Japan) attached to a triple quadrupole MS (Shimadzu). To quantify beany off-flavor compounds, their standard curves were generated from PBMA patties spiked to contain 0.1-, 0.5–1.0-, 5.0-, and 10-μg standard/g-patty for volatile compounds.

### In vitro digestion

*In vitro* digestion was conducted using the INFOGEST digestion method [32]. To simulate the oral phase, 5 mL of simulated saliva amylase activity (75 U/mL) in the final oral mixture (pH 7.0) was added to 5 g of finely crushed PBMA patties. The mixture was agitated for 2 min to mimic mastication in a human mouth. To simulate the gastric phase, simulated gastric juice containing pepsin activity (2,000 U/mL) in the final stomach mixture was added to the oral phase sample (volume ratio 1:1), after which the mixture was adjusted to pH 3.0. Protein digestion in the simulated stomach was conducted at pH 3.0 for 2 h. To simulate the intestinal phase, 20 mL of a simulated intestinal juice containing trypsin activity (100 U/mL) and lipase activity (2,000 U/mL) was added to the stomach phase sample, after which the mixture was adjusted to pH 7.0. Protein digestion in the intestine was conducted for 2 h. Samples from all three phases were boiled at 100°C for 5 min. Free amino nitrogen content was measured using the ninhydrin method.

### Statistical analysis

SPI and TVP in three different lots were purchased from the manufacturer. Data are presented as the mean ± standard error of five independent experiments to evaluate the effects of different sample formulations and preparations. Tukey’s test at a significance level of 95% (*p*<0.05) and 99% (*p*<0.01) was applied to determine the significant differences in the results.

## Results and discussion

### PG-catalyzed deamidation of SPI and TVP

**Fig 1:**
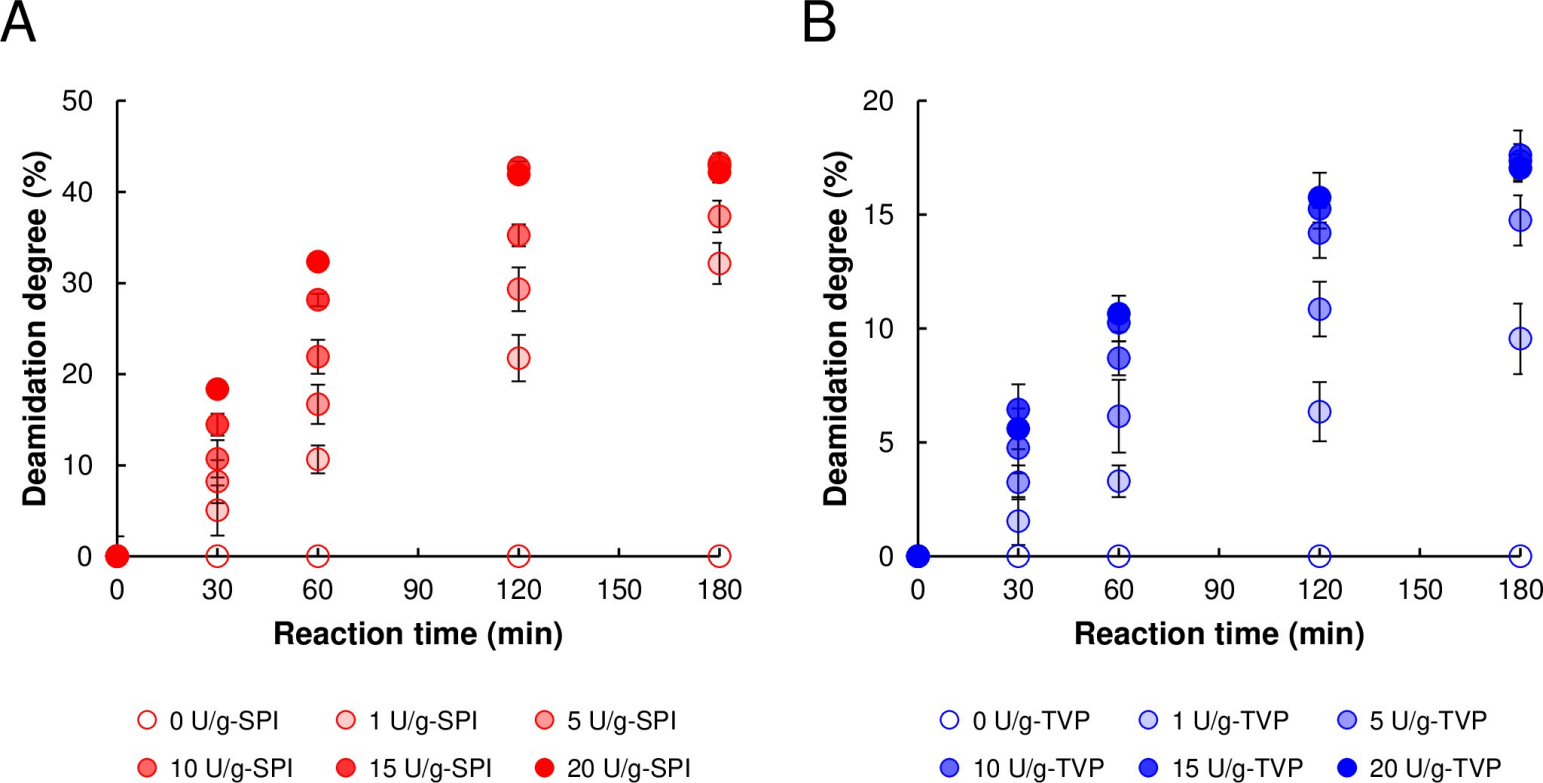
Time-course of PG-catalyzed deamidation of soy protein isolate (SPI) and textured vegetable proteins (TVP). SPI (A) and TVP (B) were treated with 0–20 U/g-protein. Error bars represent the mean ± standard error of five independent experiments.

The time-course of the PG-catalyzed deamidation of SPI and TVP was investigated. Fig 1 shows the DD of SPI and TVP after PG treatment. For SPI, the DD by 5–20 U/g-protein PG increased in a time-dependent manner and plateaued at 45% after a 180-min reaction, similar to the 42% DD reported by Jiang et al. (2022) [33] and 50% reported by Suppavorasatit et al. (2011) [34]. For TVP, the DD by 10–20 U/g-protein PG increased and plateaued at 17.0% under the same conditions; this is because the particle size of TVP is much larger than that of SPI, which reduces the surface area for attack by PG. Moreover, a previous study reported that heat pretreatment can reduce PG deamidation activity [33], which may be because excessive thermal treatment of soy proteins induces highly aggregated proteins, leading to some Gln residues embedding in the aggregates [33]. TVP products are also commonly texturized by pressing them at high temperatures and pressures [11], resulting in similar folding of Gln residues inside the TVP. Therefore, it was suggested that the deamidation activity of the TVP was lower than that of the SPI.

### Effects of PG on the physical properties of SPI

To clarify the physical properties of TVP deamidated by PG in detail, we first investigated the properties of the deamidated SPI (Table 1). Although both the deamidated and control proteins were not hydrolyzed, the solubility of deamidated proteins was 2.17-fold higher than that of the control proteins. Generally, protein solubility is an industrially important indicator of the performance of protein functionalities, such as liquid-holding capacities, and gelling, foaming, and emulsifying properties. Liquid-holding capacities are important factors for food juiciness. Surprisingly, the water- and oil-holding capacities of deamidated proteins improved by 1.56- and 1.53-fold, respectively compared to those of control proteins. Foaming properties (foaming capacity and foam stability) are an important factor in the processing of creamy (dairy) products. The foaming capacity of deamidated proteins was significantly enhanced (2.92-fold), and foam stability was enhanced (1.04-fold) without significant difference, compared to that of control proteins. Emulsifying properties (emulsifying activity and stability) are important factors in suppressing oil leakage and improving the mouthfeel of foods. Both emulsifying activity and stability of deamidated proteins improved significantly by 5.5- and 1.6-fold, respectively. These findings suggest that PG-catalyzed deamidation improved the physical properties and usefulness of soy-based protein products.

**Table 1:** Physical properties of protein-glutaminase (PG)-treated and control soy protein isolate (SPI).

	Control SPI	PG-treated SPI
Degree of hydrolysis (%)	0	0
Solubility (%)	24.2±3.7^b^	52.4±6.2^a^
Water-holding capacity (g/g-protein)	2.5±0.3^b^	3.9±0.4^a^
Oil-holding capacity (g/g-protein)	3.2±0.3^b^	4.9±0.3^a^
Foaming capacity (%)	7.3±1.2^b^	21.3±1.3^a^
Foam stability (%)	67.4±1.0^a^	69.9±1.1^a^
Emulsifying activity index (m^2^/g)	4.4±1.6^b^	24.1±0.4^a^
Emulsifying stability index (%)	54.4±6.4^b^	86.7±7.9^a^

Data are presented as mean ± standard error of five experiments. Different letters in the same row indicate significant differences (*p*<0.05).

Generally, plant-derived proteins have high insoluble glutamine content, leading to the aggregation and precipitation of proteins via hydrophobic interaction or hydrogen bonds. In these proteins, PG catalyzes the deamidation of glutamine residues to glutamic acid residues. Notably, PG-catalyzed deamidation can increase the number of negatively charged glutamic acid residues in protein molecules [35]. In previous studies, it was reported that PG treatment enhanced the solubility of plant-derived proteins such as oat, soy, pea, wheat, or coconut proteins in a time-dependent manner [17, 34, 36–38]. This is because increasing negative charges of protein structure by PG increases the electrostatic repulsion, thereby weakening the ability to aggregate and precipitate intra- and intermolecular proteins and enhancing the protein solubility in water [35]. Similarly, enhanced solubility owing to PG deamidation improves the emulsifying and foaming properties of plant-derived proteins such as soy, wheat, zein, oat, and coconut [17, 34, 36–38]. This is because newly introduced negative charges to proteins by PG, unfolds their structure and exposes their hydrophobic regions to provide proteins with an amphiphilic property [35]. Thereafter, deamidated proteins can adapt to a polar-nonpolar interface [35].

### Effects of PG on the physical properties of TVP

**Fig 2:**
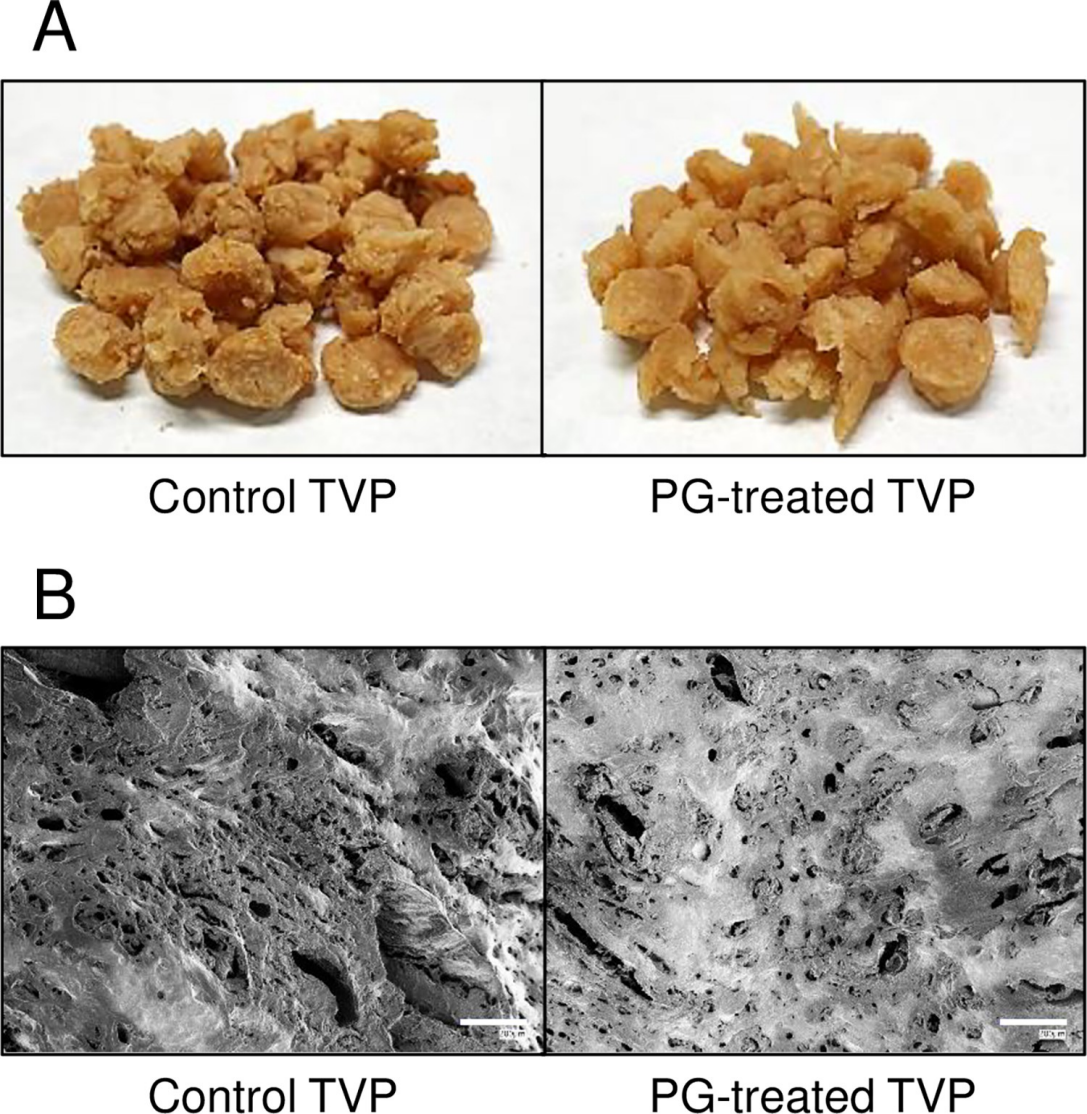
The appearance of protein-glutaminase (PG)-treated and control TVP. PG-treated and control TVP were evaluated based on their appearance (A) and scanning electron microscope (SEM) images (B). Scale bar = 200 μm.

**Fig 3:**
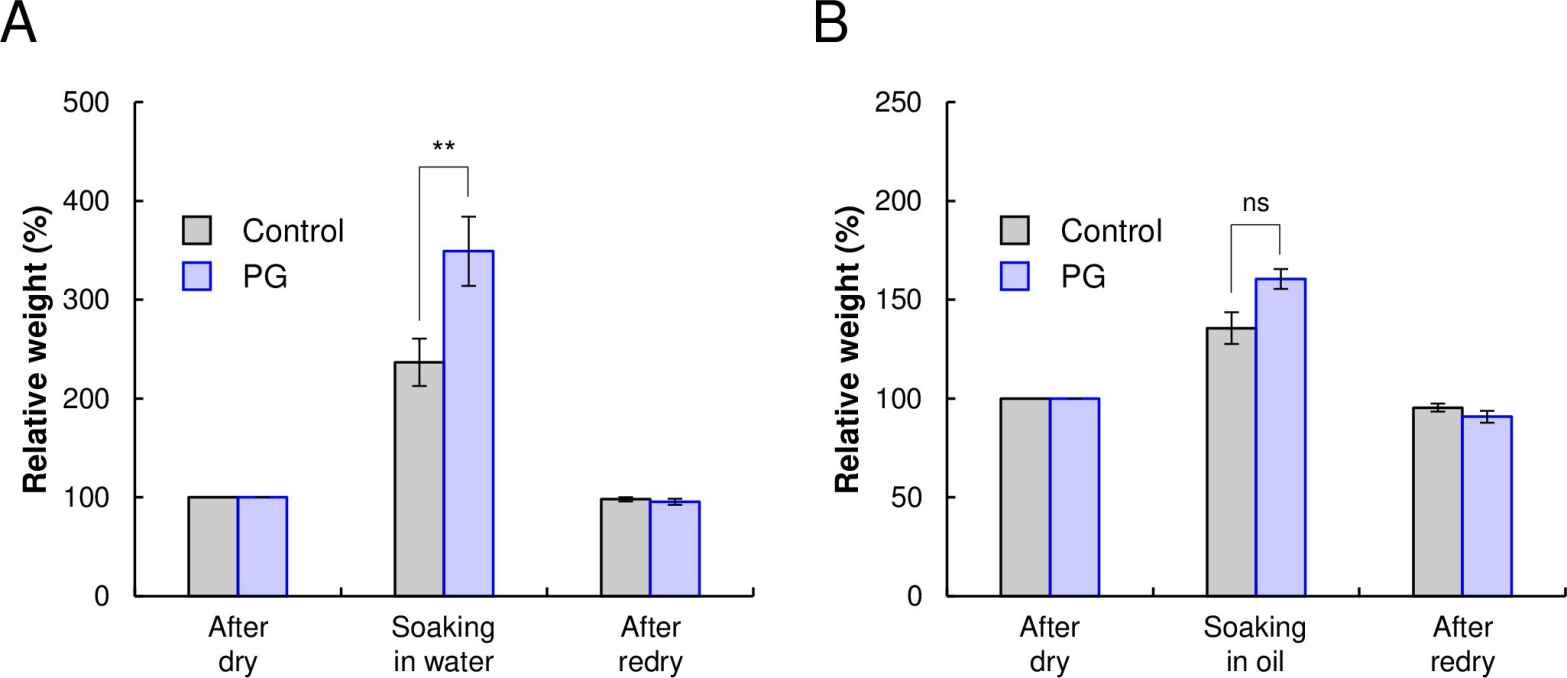
Water- and oil-holding capacity of PG-treated and control TVP. The water- (A) and oil-holding capacities (B) of the PG-treated and control TVP. Dried TVP was soaked in water or canola oil and their weights were measured.

Fig 2 shows the appearance and SEM images of the control and PG-treated TVPs after hydration. Visually, there was no significant difference between the control and PG-treated TVP, but the latter was slightly more hydrated (Fig 2A). However, the SEM analysis revealed a significant difference between the control and PG-treated TVP (Fig 2B). Interestingly, the PG-treated TVP had a rounded shape, whereas the control TVP had cut surfaces because of the manufacturing process. Having confirmed the visual differences in the TVPs, we then evaluated their physical properties. As shown in Fig 2A, the PG-treated TVP exhibited slightly increased hydration. Therefore, after the control and PG-treated TVP were dried, their ability to absorb water and oil was examined (Fig 3). The ability of the PG-treated TVP to absorb water was improved by 1.47-fold compared to that of the control (Fig 3A), whereas the ability of PG-treated TVP to absorb oil was slightly higher than that of control (Fig 3B). These profiles were similar to those of deamidated SPI (Table 1). Moreover, the relative weights of the TVPs were maintained after redrying. These results indicate that PG treatment enhanced the ability of TVP to absorb water and oil without hydrolysis of the protein chain.

### Physical properties of PBMA patties composed of PG-treated TVP

**Fig 4:**
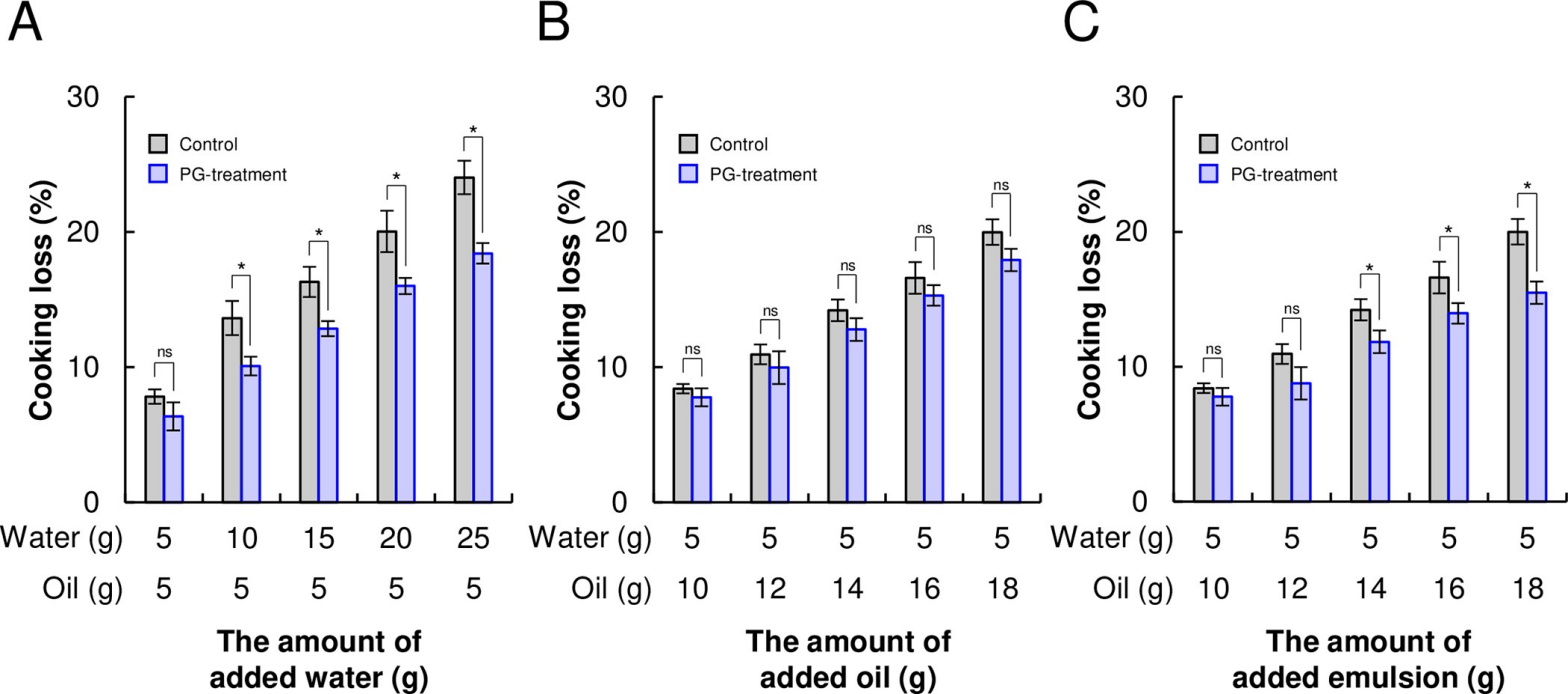
Cooking loss values of plant-based meat analog (PBMA) patties. Cooking loss values after grilling PBMA patties containing different amounts of water (A), oil (B), and emulsions (C).

The increased water and oil absorption ability due to PG treatment might increase the juiciness of PBMA patties composed of PG-treated TVP. Cooking loss was evaluated as an indicator of juiciness in this study. For PBMA patties, the cooking loss value shows product shrinkage degree or yields during cooking. We evaluated cooking losses by adding different amounts of water and canola oil to the recipe for PBMA patties. As the amounts of water and oil in the patties increased, cooking loss increased in a dose-dependent manner (Fig 4). Surprisingly, the water loss during cooking of the PBMA patties composed of PG-treated TVP was substantially lower than that of the control patties. In contrast, the oil loss during cooking of the PBMA patties composed of PG-treated TVP was slightly lower than that of the control patties, although the difference was not significant (*p*>0.05). When various amounts of water and oil were added, the water and oil loss during cooking of the PBMA patties composed of PG-treated TVP decreased by 1.5–5.6% and 0.7–2.6% compared with control PBMA patties, respectively. Table 1 shows that the PG-treated proteins had a much higher emulsifying ability than the control. Therefore, water and oil emulsions were added to the PBMA patties. Interestingly, the emulsion loss of PBMA patties composed of PG-treated TVP decreased by 4.5% and 2.5% compared to control PBMA patties (Fig 4C) and PBMA patties composed of PG-treated TVP (Fig 4B). These findings indicate that the PG treatment of TVP could contribute to the water- and emulsion-holding capacity of PBMA patties, potentially leading to a juicy texture.

The improved cooking loss values of the PBMA patties composed of PG-treated TVP (Fig 4) might be because liquid-holding capacities of SPI and TVP are enhanced after PG treatment (Table 1 and Fig 3). In previous studies, circular dichroism spectroscopy showed that PG-catalyzed deamidation weakens intra- and intermolecular hydrogen bonds between proteins, thereby unfolding protein structures and exposing hydrophilic regions within the protein [34, 37, 38]. Thus, more hydrogen bonds can form between water molecules and hydroxyl groups of exposed proteins deamidated by PG [39], possibly leading to an increase in the water-holding capacity of the deamidated proteins. Moreover, in several studies, PG-catalyzed deamidation unfolded and exposed the hydrophobic regions of proteins [33, 40, 41]. These exposed hydrophobic regions in deamidated proteins have a higher affinity for lipid molecules [26], which enhances the oil-holding capacity of deamidated proteins; thus, holding capacities of soy proteins deamidated by PG may be induced by unfolding and exposing the protein structures, and forming new hydrogen bonds with water molecules and hydrophobic interactions with oil molecules. Similar to SPI, enhancement of water- and oil-holding capacities of protein by PG treatment may improve the cooking loss values of PBMA patties composed of PG-treated TVP.

We also investigated the color and physical properties of PBMA patties containing deamidated TVP. First, the physical properties of deamidated TVP-based patties were examined (Table 2). Interestingly, the control PBMA patties showed a higher hardness than those composed of deamidated TVP. Cohesiveness and springiness were significantly higher in PBMA patties composed of deamidated TVP compared to the control. Both patties had similar chewiness values. The effects of PG treatment on the color properties of the grilled PBMA patties were measured using a colorimeter (Table 2). Compared to the control PBMA patties, the L* value of the PBMA patties composed of deamidated TVP was significantly higher. In previous studies, an increase in water and/or oil content in PBMA patties led to increased L* values because small spherical water droplets and oil reflect more light [19, 30, 42–44]. Therefore, the higher water- and oil-holding capacity of deamidated TVP improves cooking loss, suggesting an increase in the L* value for PBMA patties composed of deamidated TVP. The a* and b* values of the PBMA patties composed of deamidated TVP were similar to those of the control. These findings indicate that the deamidated TVP-based PBMA patties were more vivid in color without changing the colors.

**Table 2:** Physical and objective color measurements of PBMA patties.

Treatment	Control patty	Patty composed of PG-treated TVP
Hardness (N)	20.7±1.1^a^	17.2±0.6^b^
Cohesiveness	0.72±0.04^b^	0.86±0.06^a^
Springiness	0.69±0.02^b^	0.82±0.09^a^
Chewiness (N)	10.2±1.2^a^	12.1±1.5^a^
L* value	49.8±1.2^b^	54.1±0.9^a^
a* value	5.7±0.4^a^	6.1±0.7^a^
b* value	13.2±1.1^a^	12.6±0.6^a^

Data are presented as the mean ± standard error of five experiments.

L*, lightness

a*, redness

b*, yellowness. Different letters in the same row indicate significant differences (*p*<0.05).

### Beany off-flavor of PBMA patties composed of PG-treated TVP

Notably, the PG-treated TVP had a stronger beany off-flavor than the control TVP based on the experimenter’s sensory perception, which was easily removed by washing. Based on previous studies, beany off-flavor is caused by a mix of numerous volatile compounds derived from fatty acids and their derivates during the growth and processing of soybeans [45, 46]. Among them, carbonyl compounds (e.g., hexanal), fatty alcohols (e.g., 1-octen-3-ol), and aromatic compounds (e.g., benzaldehyde) are considered to contribute to the off-flavor [46]. Many methods are currently being used to remove the compounds causing the off-flavor, such as breeding, fermentation, and physical, chemical, and genetic engineering methods [47]. However, these methods are insufficient to treat the technical challenges related to eliminating beany off-flavors. This unpleasant flavor due to volatiles from soybean protein-related ingredients limits the consumer acceptability of PBMA products [48]. Thus, the effects of PG and the wash process on beany off-flavor compounds volatilized from PBMA patties were investigated using HS-SPME-GC/MS.

**Fig 5:**
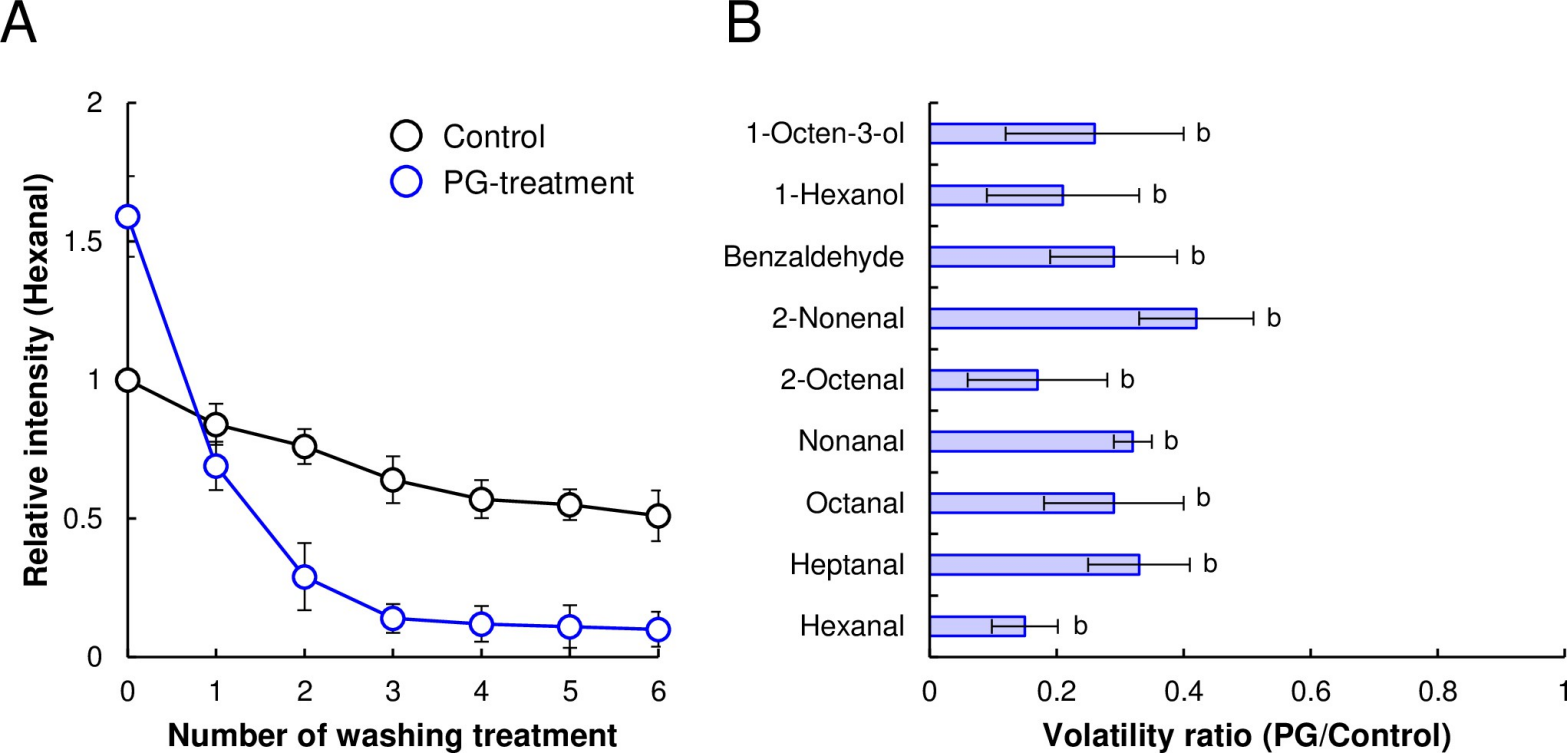
Analysis of beany off-flavor compounds in PBMA patties using S-HPME-GC/MS. (A) Relative intensity of n-hexanal released from PBMA patties washed multiple times. (B) Volatility ratio of beany off-flavor compounds released from PBMA patties after three washes. Different letters indicate significant differences (*p*<0.05).

PG-treated and control TVPs were washed multiple times, and PBMA patties composed of each TVP were prepared. After grilling process, the residual beany off-flavor compounds in the PBMA patties were quantified (Fig 5). The amount of volatile hexanal from PBMA patties composed of PG-treated TVP without washing was 1.59-fold higher than that of the control PBMA patties (Fig 5A). As the number of washes increased, the amount of hexanal volatilized from the control PBMA patties gradually decreased and plateaued at a 49% reduction. The volatile content of the PBMA patties composed of PG-treated TVP sharply decreased to 10% and reached a plateau after the third wash. We also examined the amount of other beany off-flavor compounds released from the PBMA patties containing TVPs after the third washing process. Similar to hexanal, the volatile amounts of fatty aldehydes, such as hexanal, heptanal, octanal, nonanal, 2-octenal, 2-nonenal, and benzaldehyde, and fatty alcohols, such as 1-hexanol and 1-octen-3-ol decreased between 14 and 42% (Fig 5B). These findings indicate that PG treatment for TVP can easily remove various beany off-flavor compounds by washing.

In general, flavor compounds and protein molecules interact non-covalently and covalently. The non-covalent interactions include hydrogen bonds, hydrophobic interactions, electrostatic interactions, and van der Waals force [49]. Whereas the covalent interaction includes Schiff bases formed between carbonyl group-containing flavor compounds (e.g., aldehydes and ketones) and proteins at their amide side chains (e.g., glutamine and asparagine residues) [50]. Some flavor and plant protein interactions can be reduced by PG treatment [34, 36, 51]. Plant-derived proteins such as soy and coconut deamidated by PG reduced the ability of binding hydrophobic carbonyl-containing compounds vanillin and maltol [34, 36, 51]. The decrease in the flavor-binding affinities of proteins deamidated by PG may be due to the introduction of a negative charge (glutamic acid residues), which leads to the replacement of stronger hydrophobic interactions or Schiff base formations to weaker van der Waals forces or hydrogen bonds [34, 36, 51]. Therefore, PG treatment could contribute to reducing undesirable flavors in soy-based foods.

### In vitro digestibility of PBMA patties

**Fig 6:**
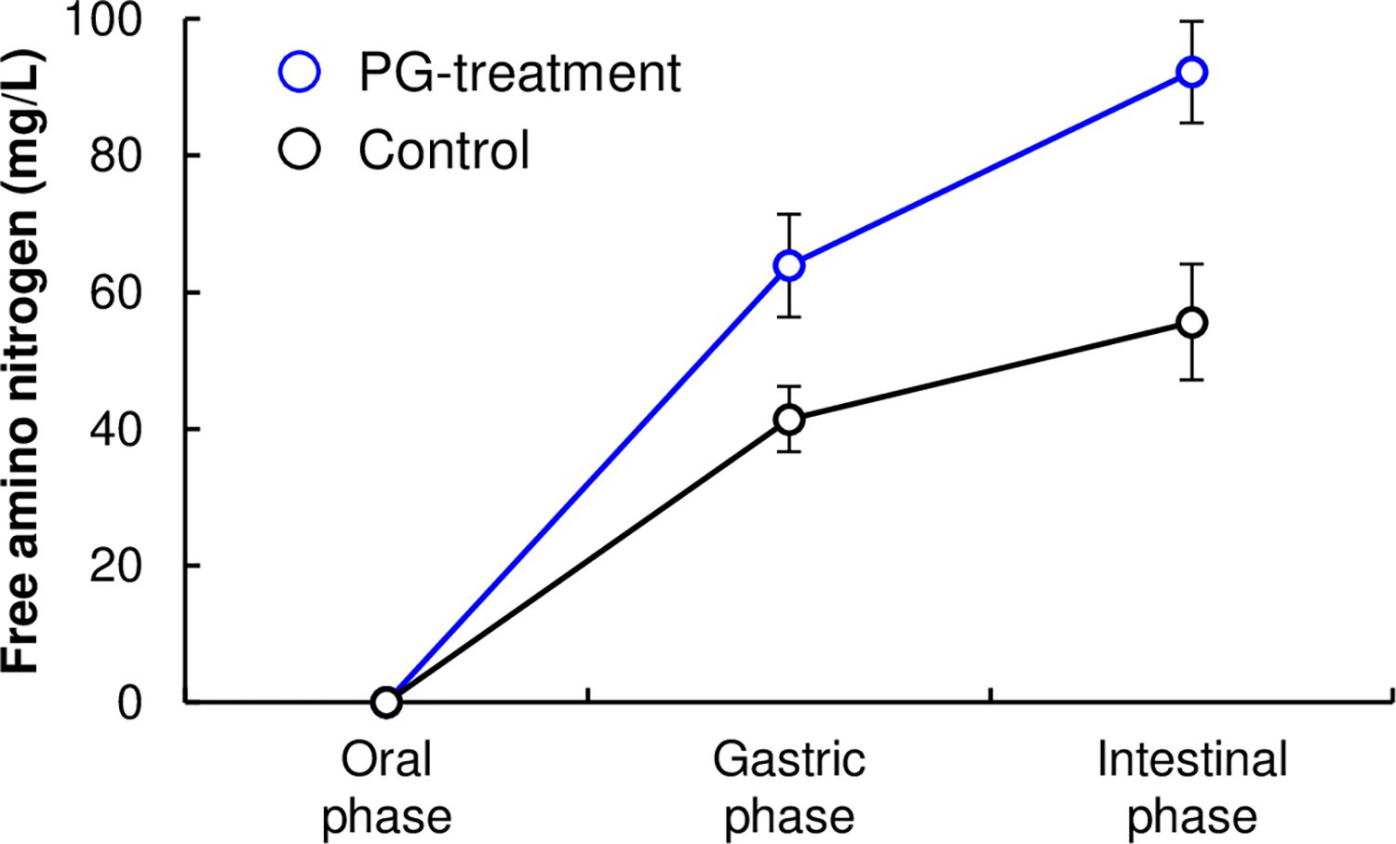
*In vitro* digestion test of PBMA patties. The amount of free amino nitrogen (FAN) released from PBMA patties after intestinal digestion. Error bars represent the mean ± standard error of three independent experiments.

Fig 6 shows free amino nitrogen (FAN) released from the PBMA patties after *in vitro* digestion (INGOGEST method). The amounts of FAN released from PBMA patties containing PG-treated TVP were 1.5- and 1.7-fold higher than those released from control patties in the gastric and intestinal phases. This indicates that PBMA patties composed of PG-treated TVP were easier to degrade with digestive enzymes and absorb nutrients. This enhancement was attributed to its higher cohesiveness, springiness, and water-holding capacity (Fig 4 and Table 2). Cohesiveness and springiness values display a degree of gel that has greater resistance to a second deformation compared with the first deformation. Generally, foods with higher water content have lower fragment density after mastication in the mouth than foods with lower water contents [29]. Therefore, it is suggested that the dispersibility of PBMA patties composed of PG-treated TVP in the simulated gastric and intestinal phases was higher, thereby enhancing digestibility of the patties.

## Conclusions

We performed enzymatic deamidation of TVP (the main ingredient) using PG to enhance the water- and oil-holding capacities of PBMA patties or TVP while reducing food additives. PG treatment enhanced the liquid-holding capacities (juiciness indicator) of PBMA patties and TVP. Interestingly, PG treatment of TVP could easily remove various beany off-flavor compounds by washing, reducing them by 58–85%. Furthermore, the amounts of FAN (indicator of protein digestibility) were 1.5- and 1.7-fold greater than control patties in the gastric and intestinal phases, respectively. Because PG is inactive after grilling process, it is not an additive by regulation. Our findings indicate that enhancing the juiciness of PBMA patties using PG might be a new strategy for meeting new clean-label requirements. This strategy could help food manufacturers develop clean-label PBMAs that are more attractive to consumers.
